# Proposed method of histological separation between connective tissue disease-associated interstitial pneumonia and idiopathic interstitial pneumonias

**DOI:** 10.1371/journal.pone.0206186

**Published:** 2018-11-05

**Authors:** Mutsumi Ozasa, Hiromi Ichikawa, Shuntaro Sato, Tomonori Tanaka, Takeshi Johkoh, Kensuke Kataoka, Yasuhiko Yamano, Yasuhiro Kondoh, Hideki Nakamura, Atsushi Kawakami, Andrey Bychkov, Hiroyuki Taniguchi, Junya Fukuoka

**Affiliations:** 1 Nagasaki University Hospital, Nagasaki Educational and Diagnostic Center of Pathology (NEDCP), Nagasaki, Japan; 2 Clinical Research Center, Nagasaki University Hospital, Nagasaki, Japan; 3 Department of Radiology, Kinki Central Hospital of Mutual Aid Association of Public School Teachers, Itami, Hyogo, Japan; 4 Department of Respiratory Medicine and Allergy, Tosei General Hospital, Seto, Aichi, Japan; 5 Unit of Translational Medicine, Department of Immunology and Rheumatology, Nagasaki University Graduate School of Biomedical Sciences, Nagasaki, Japan; 6 Department of Pathology, Kameda Medical Center, Kamogawa, Japan; Toranomon Hospital, JAPAN

## Abstract

**Objectives:**

Idiopathic interstitial pneumonia (IIP) and connective tissue disease -associated interstitial pneumonia (CTD-IP) are the two most common types of interstitial pneumonia. IIP and CTD-IP share common histological features, yet their clinical management is different. Separation of the two conditions based solely on histology can be challenging, and there are no established criteria.

**Materials and methods:**

We selected 105 consecutive cases of IIP (79 usual interstitial pneumonia and 26 non-specific interstitial pneumonia) and 49 cases of CTD-IP for derivation and 32 cases of IIP and 10 cases of CTD-IP for validation. Fourteen histological parameters were evaluated independently by two pathologists for derivation group and graded into 0 to 3. The association between the score for each marker and a diagnosis of CTD was investigated using Fisher’s exact test and stepwise logistic regression analysis. A formula for calculating the probability of IIP and CTD-IP was constructed by the markers identified in the regression test with coefficients for each finding. The formula was confirmed using validation case group.

**Results:**

Stepwise logistic regression analysis showed that plasmacytosis, lymphoid follicle with germinal center, and airspace fibrin were suggestive of CTD-IP and that fibroblastic foci, smooth muscle hyperplasia, cellular IP, dense perivascular collagen, and fat metaplasia were suggestive of IIP. The formula used to calculate the probabilities based on estimated values for each finding was created, and user-friendly web based app was composed at www.ctdip.com.

On the validation study, 30 out of 32 IIP and eight out of 10 CTD-IPs were distinguished correctly by the app (Specificity: 93%, Sensitivity: 80%).

**Conclusions:**

We identified histological markers and derived a practical formula and user-friendly app to distinguish CTD-IPs from IIP.

## Introduction

Interstitial pneumonia (IP) is a heterogeneous group of parenchymal lung disorders of variable aetiology. The most common aetiological types are idiopathic IPs (IIP) and connective tissue disease-associated IP (CTD-IP). They are considered to be distinct conditions, but share common radiologic, pathologic, and clinical features[1], and the distinction can be challenging even after multidisciplinary discussion (MDD) by experienced pulmonary experts[2]. User-friendly criteria to separate these two conditions are needed.

Several studies showed that patients with CTD-IP have a better prognosis than those with IIP[3–6], and the current recommendations for clinical management of the two conditions are markedly different[7, 8]. Therefore, an accurate distinction between IIP and CTD-IP is critical for being able to treat patients appropriately. A recent publication suggests that IIP with autoimmune features have similarities with CTD-IP[9]. The majority of cases present with IP after development of systemic CTD; however, the status of CTD is uncertain in a significant number of cases when lung disease presents as an initial symptom. Also as a realty, for majority cases, consultation to rheumatologist is not available. In such cases, separation of the two conditions is challenging, and then, pathologists feel difficulty to distinguish because of the lack of established histological criteria.

Histological features suggestive of CTD based on expert opinion have been reported in a few textbooks[10, 11]. From those references, Fischer et al. have put forward the concept of lung dominant CTD, in which they proposed four histological markers without high evidence: extensive pleuritis, lymphoid aggregates with germinal centre, prominent plasmacytic infiltration, and dense perivascular collagen[12]. A distinct entity known as IP with autoimmune features (IPAF) has been further published by the American Thoracic Society/European Respiratory Society and includes histological factors such as lymphoid aggregates with germinal centre and diffuse lymphoplasmacytic infiltration in a morphological domain[13]. However, these lists of criteria still have been suggested based on expert opinions and their significance as markers has not been well validated. Recently proposed criteria of IPF by Fleischner society also stated that the evidence is not enough to separate IPAF from IIP[14]. Therefore, we sought to investigate the pathological differences between IIP and CTD-IP, with the aim of useful and reproducible data for day-to-day diagnosis.

The aim of this study was to semi-quantitatively evaluate histological findings in patients with chronic fibrotic IP to identify markers suggestive of CTD, derive a practical formula, and create a user friendly web-based application for routine use in a clinical practice.

## Materials and methods

This retrospective investigation was approved by Ethical committee of Nagasaki University Hospital (protocol 14012746).

### Case selection for derivation and validation cohorts

For derivation cases, we reviewed video-assisted thoracoscopy (VATS) biopsies diagnosed by MDD as chronic fibrotic IP in the category of IIP and CTD-IP at a single institution between 2008 and 2013. Clinical data including age, sex, smoking history, presence of autoantibodies and other relevant antigens, use of feather products, occupational history, and the results of pulmonary function tests were obtained from patients’ medical records.

For validation cases, VATS biopsies showing fibrotic IP and being diagnosed as IIP or CTD-IP at the same institute between 2014 and 2015 were enrolled. The diagnosis of CTD was made according to the following criteria of rheumatoid arthritis (RA)[15], systemic sclerosis (SSc)[16], mixed connective tissue disease (MCTD)[17], polymyositis/dermatomyositis (PM/DM)[18], and Sjogren’s syndrome (SjS)[19]. The histological patterns of the cases were determined by consensus between two experienced pulmonary pathologists. To limit to the cases of chronic fibrosing IPs, cases with only Usual IP (UIP) and Non-specific IP (NSIP) patterns were included for the analysis. The cases of IIP with clinical IPAF domains were excluded due to the following reasons. First, the concept of IPAF is still in debate and a use of this terminology is limited to research purposes. Second, the evaluation of IPAF cases would increase uncertainty for our research design.

### Pathological evaluation for derivation cases

In each patient, the VATS specimens were taken from one to three lobes. The slides were reviewed independently by two pathologists who were blinded to the clinical data and diagnosis. The level of each finding was graded as 0 (none), 1 (mild), 2 (moderate), and 3 (marked) for 14 histological features. The histological features scored were honeycombing (HC), fibroblastic focus (FF), smooth muscle hyperplasia (SMH), organising pneumonia (OP), cellular interstitial pneumonia (CIP), prominent plasmacytic infiltration (Plasm), lymphoid follicle with germinal centre (LyGC), extensive pleuritis (PLE), vascular intimal thickening (VT), dense perivascular collagen (DPVC), airspace fibrin (AF), fat metaplasia (Fat), constrictive bronchiolitis (CB), and bronchiectasis (S1 Fig). All evaluations were performed using haematoxylin-eosin stained whole slide images obtained at 200× magnifications by scanners (Aperio CS, Sausalito, CA, USA; VS100, Olympus, Tokyo, Japan). For each findings, concordant cases were defined to include cases with identical scores and scores with 1 level of difference between the two pathologists. The average values for these concordant scores were used for further analysis. Scores that met more than 1 level of difference between the two pathologists were considered to be discordant, and the consensus scores were discussed further with another experienced pulmonary pathologist.

### Statistical analysis

Fourteen scored features along with the histopathological diagnosis, UIP versus NSIP, were compared between IIP and CTD-IP. Simple logistic regression analysis was used to identify markers indicative of both IIP and CTD-IP. Receiver-operating characteristic (ROC) curves were constructed for each feature. Stepwise logistic regression analysis was then used to select useful markers and generate a formula to calculate the probability of IIP. A ROC curve was constructed to validate the formula. Kaplan Meier curve was plotted for survival analysis. All statistical analyses were performed by a statistician using R version 0.99.902 (The R Foundation for Statistical Computing, Vienna, Austria).

### Developing of web-based application

To make it more practical for pathologists and/or pulmonologists to use the formula, web-based application was created at www.ctdip.com. The app lists eight queries of indicated morphometrical markers and manipulate the calculated answer with its likelihood of the diagnosis based on the probability: likelihood A, probability from1.0 to 0.8 and 0.19 to 0; likelihood B, 0.79 to 0.6 and 0.39 to 0.2; likelihood C, 0.59 to 0.4, respectively. The probability is generated by allocating a score to each feature with a range from 0 to 1. Probability > 0.5 was indicated that the case was more suggestive to be CTD-IP.

### Application of the formula to validation cases

Two pathologists evaluated validation cases and consensus score for the eight findings were extracted. The scores were applied to the APP, the cases were separated based on the results of APP, and its specificity and sensitivity were evaluated.

## Results

**Fig 1:**
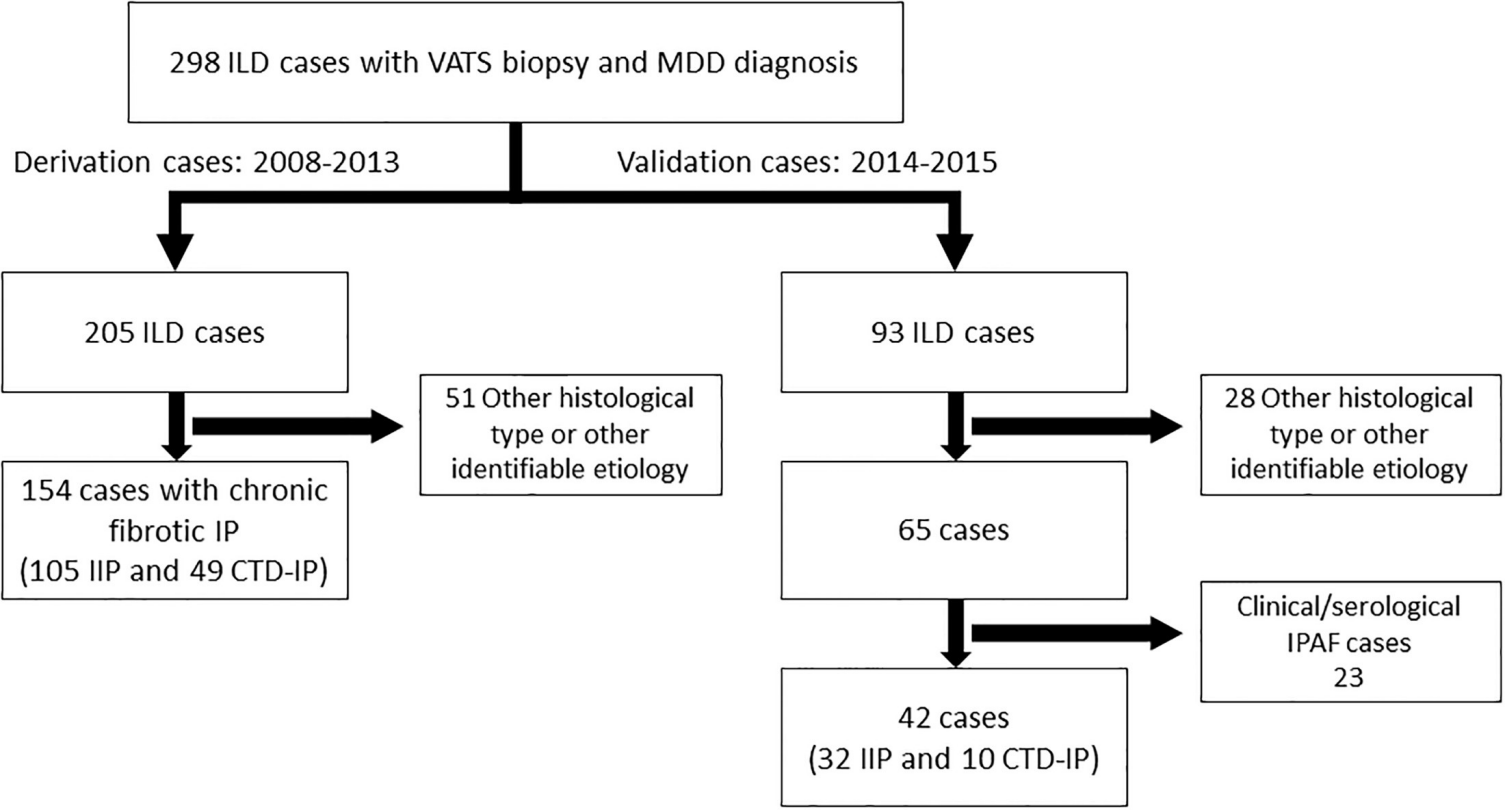
The CONSORT flow diagram. ILD: interstitial lung disease; VATS: video associated thoracic surgery; MDD: multidisciplinary discussion; IP: interstitial pneumonia; IIP: idiopathic interstitial pneumonia; CTD-IP: connective tissue disease associated interstitial pneumonia; IPAF: interstitial pneumonia with autoimmune features.

The review identified 154 out of 205 cases for derivation that included 105 IIP and 49 CTD-IP (Fig 1). The 49 CTD-IP developed in 20 patients with rheumatoid arthritis, 12 with systemic sclerosis, 8 with polymyositis/dermatomyositis, 6 with Sjögren’s syndrome, and 3 with mixed CTD. The histological patterns were UIP (n = 79) and NSIP (n = 26) in the IIP group, and UIP (n = 19) and NSIP (n = 30) in the CTD-IP group. Eleven patients in each group were on steroid therapy at the time of VATS. None of the patients received chemotherapy or radiation before VATS. The clinical characteristics of the patients are shown in Table 1, S1 Table, and S2 Fig.

**Table 1:** Patient characteristics.

Variable	IIP(n = 105)	CTD-IP(n = 49)	*P* -value
Age	63±8.2	60±8.7	0.03
Sex			
Female	32(30%)	30(61%)	<0.01
Male	73(70%)	19(39%)	
Smoking history			
Current smoker	4(4%)	3(6%)	0.04
Ex-smoker	67(64%)	21(43%)	
Never smoker	34(33%)	25(51%)	
ANA*	12(11%)	21(43%)	<0.01
KL-6	1381±959	2441±6242	NS
%FVC	84.6±21.5	79.5±23.3	NS
%DLCO	62±20	54±15	0.01

*ANA ≧ 320 was considered positive

For validation cases, out of 93, 42 cases were identified to have chronic fibrotic IP with either idiopathic or CTD conditions. IIP with clinical IPAF features were not included due to its uncertainty. H&E slides were reviewed by two pathologists and consensus score for each findings were extracted and applied to the formula by using web-based application (www.ctdip.com). 10 out of 42 cases had definite CTD: 3 SSc, 3Sjogren, 2 RA, and 2 PM/DM.

Patients with CTD-IP were younger (*P* = 0.03) than those with IIP. The CTD-IP group contained significantly more female patients (*P*<0.01) and serum anti-nuclear antibody titres were significantly higher in this group (*P*<0.01) than in the IIP group. Patients with IIP had higher exposure to tobacco smoking (*P* = 0.04). Pulmonary function tests were almost identical in the two groups except that the percent predicted diffusing capacity for carbon monoxide was significantly lower in patients with CTD-IP (*P* = 0.01). Other serum markers, including KL-6, and pulmonary function tests, including percent predicted forced vital capacity, were not significantly different between the two groups.

**Fig 2:**
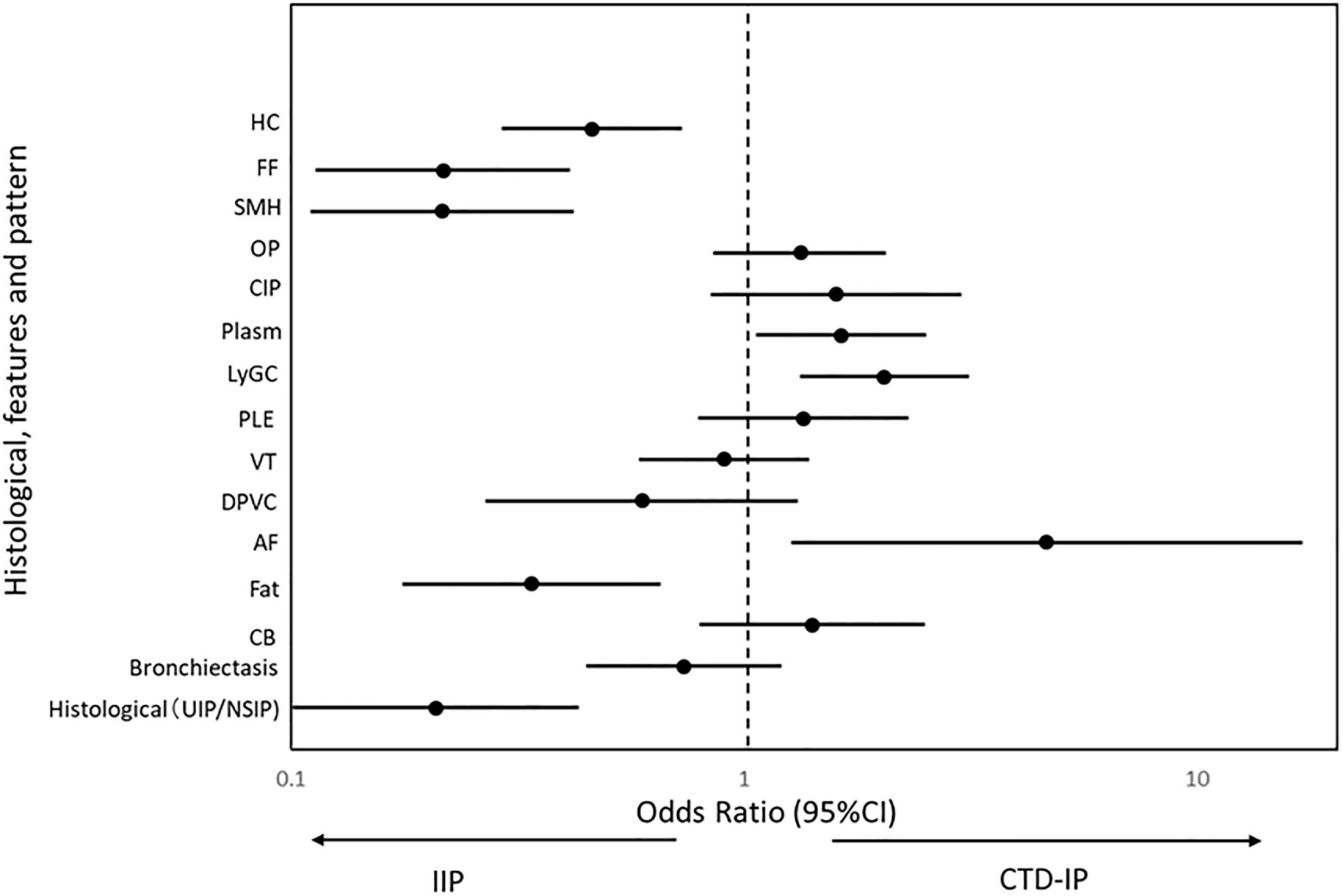
Odds ratio comparing connective tissue disease associated interstitial pneumonia and idiopathic interstitial pneumonia. Eight out of 14 scored histological findings along with histological diagnosis of usual interstitial pneumonia (UIP) versus nonspecific interstitial pneumonia (NSIP) are identified by simple Logistic regression to separate idiopathic interstitial pneumonia (IIP) and connective tissue disease associated interstitial pneumonia (CTD-IP).

The inter-observer agreement with regard to the histological findings was good for LyGC (κ = 0.67) and SMH (κ = 0.63), and moderate for HC (κ = 0.58) and Fat (κ = 0.45), fair for bronchiectasis (κ = 0.39), constrictive bronchiolitis (κ = 0.37), CIP (κ = 0.26), vascular intimal thickening (κ = 0.24) and FF (κ = 0.23), and poor for Plasm (κ = 0.14), AF (κ = 0.14), DPVC (κ = 0.1), PLE (κ = 0.07), and OP (κ = 0.03). Simple logistic regression analysis showed that HC, FF, SMH, vascular intimal thickening, DPVC, Fat, bronchiectasis and a histological diagnosis of UIP were features indicative of IIP, whilst OP, CIP, Plasm, LyGC, PLE, AF and constrictive bronchiolitis were features of CTD-IP (Fig 2).

The ROC curves showed that the area under the curve for most of the features taken alone was low (S2 Fig). Among those 15 findings, stepwise logistic regression analysis showed that FF, SMH, CIP, DPVC, and Fat were suggestive of IIP and that Plasm, LyGC, and AF were suggestive of CTD-IP. The formula used to calculate the probabilities (P) based on estimated values for each finding is as follows:

P(Y=CTD-IP|Markers)=exp(Z)/1+exp(Z)

$$\begin{array}{l} \text{Z}=+1.65-1.09\left(\text{FF}\;\text{score}\right)-0.81\left(\text{SMH}\;\text{score}\right)-0.85\left(\text{CIP}\;\text{score}\right)-0.86(\text{DPVC}\\ \text{score})-0.57\left(\text{Fat}\;\text{score}\right)+0.86\left(\text{Plasm}\;\text{score}\right)+0.64\left(\text{LyGC}\;\text{score}\right)+2.47\left(\text{AF}\;\text{score}\right) \end{array}$$

**Fig 3:**
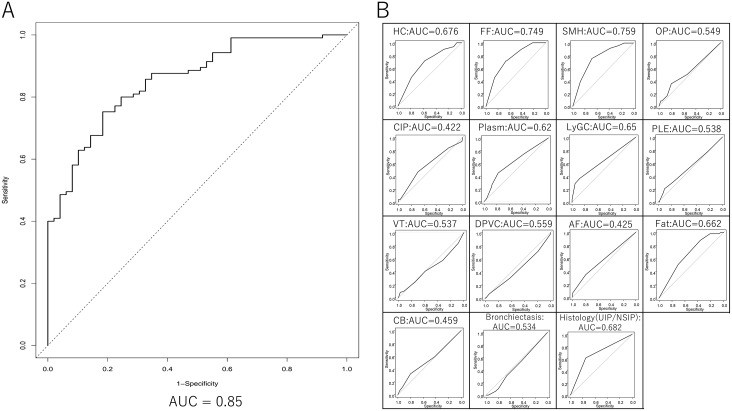
Receiver operating characteristic curve of derivation cases and 15 scored pathological factors. (A) Generated receiver operating characteristic (ROC) curve based on the formula indicates area under curve (AUC) of 0.85 for the derivation cases. (B) The area under the ROC curve for 14 findings and histological patterns, UIP and NSIP, are indicated.

The probability is generated by allocating a score to each feature. Probability > 0.5 indicated that the case was likely to be CTD-IP. A ROC curve was constructed using this formula, in which the area under the curve was 0.85 and showed the separation based on the formula is more accurate (Fig 3). Use of formula is more practical rather than using particular findings as a separation marker, since Kappa values for each finding were not enough significant. When web-based app, www.ctdip.com (S3 Fig), was used with the initial scores from two pathologists for derivation cases, the agreement of CTD-IP prediction between the two pathologists was 80% and the kappa value of 0.5.

Scores of eight findings for 42 validation cases extracted by the consensus of two pathologists were applied to the same app, www.ctdip.com, which showed sensitivity of 80% (8 of 10 CTD-IP cases) and specificity of 93% (30 of 32 IIP cases).

## Discussion

In the present study, we retrospectively analysed a large series of IP cases with VATS biopsy and identified eight histological markers to separate CTD-IP and IIP. We identified a combination of pathological findings that distinguished between CTD-IP and IIP, derived a formula to calculate the probability of being able to separate CTD-IP and IIP, and created web-based app, www.ctdip.com, which requires an input of histological scores for the eight features identified by the current study. Reasonably high specificity and sensitivity were confirmed by using the app.

Several previous studies suggested possible histological markers to separate CTD-IP from IIP[20–22]. For example, Song et al reported a diagnostic utility of GC, Plasm, FF, size of HC, and total inflammation; however, these markers were detected using univariate analysis and only LyGC remained statistically significant after adjustments by multivariate analysis. Our data confirm that some of their candidate markers (that lost significance value after multivariate analysis) were in fact independent predictors for CTD-IP. The difference in these results may be accounted for by the greater number of patients with a CTD-IP in our study. Fischer et al proposed a conceptual category of lung-dominant CTD (LD-CTD) in their report[12]. Currently, the concept of IPAF suggests a combination of morphologic parameters such as lymphoplasmacytic infiltration and lymphoid aggregates with germinal center along with histological pattern of NSIP, OP, NSIP with OP overlap, and LIP. The histological criteria they used were based on expert opinion rather than clear evidence[23]. Adegunsoye et al confirmed the significant association of histological domains in clinical IPAF cases [24]. We also expected that NSIP would be indicative of a CTD-IP; however, multivariate analysis did not yield statistical significant data.

Our stepwise logistic regression analysis detected three and five pathological findings (Plasm, LyGC and AF) indicative of CTD-IP and IIP (FF, SMH, CIP, DPVC and Fat), respectively. In this study, rather than determining the absence or presence of a single marker, we created a formula that included multiple markers to improve the diagnosis. The inter-observer agreement for each marker was not high enough, therefore use of single or a small number of markers may produce results that are poorly reproducible. Applying the coefficient based on the diagnostic value of each marker in the formula, pathologists with different levels of experience and bias can reasonably estimate the aetiology of IP. The formula itself is not practically useful unless it is converted to an easy-to-use format such as smartphone application. Of note, these eight markers are difficult to obtain with small biopsies, such as transbronchial biopsy, therefore surgical biopsy such as VATS is required for an accurate diagnosis. Recent technology of cryobiopsy may be applicable; however, due to less accumulated knowledge, its usefulness is not clear at present.

Our results can provide more objective suggestion than conventional reports which is generated solely by pathologist’s empirical opinions. And also, it may contribute to solve this common clinical dilemma and distinguish cases with underlying CTD. It will be interesting to use the app as a proposed criterion to separate patients for clinical trial.

Needless to say, CTD-IP is a quite heterogeneous group, and clinical course and treatment response are variable. Therefore, when diagnosis of CTD-IP is strongly suspected, the treatment direction for each case should be indicated after the consultation to rheumatologists.

There are several limitations in the present study. First, all the patients were Japanese. Second, we only included a small number of cases with Sjögren’s syndrome and systemic sclerosis. Third, derivation cases of IIP in this study included IIP with autoimmune features[25]. Comparison between cases of IIP with and without features of IPAF in future studies may have a clinical impact. Fourth, our case series does not include rare histological types such as desquamative interstitial pneumonia or respiratory bronchiolitis interstitial lung disease. Hypersensitivity pneumonitis (HP) is another common but challenging disease to separate. Our study excluded cases with suspected HP and focused on CTD. Similar study to separate HP may be needed as a next step.

## Conclusion

We have identified histological markers that can be used to distinguish CTD-IP from IIP. These are prominent plasmacytic infiltration, lymphoid follicle with germinal centre, and airspace fibrin for CTD-IP, and fibroblastic focus, smooth muscle hyperplasia, cellular IP, dense perivascular collagen, and fat metaplasia for IIP. We have derived a practical diagnostic formula for clinical use. The value of our data needs to be confirmed in further clinical studies with larger numbers of patients.

## Supporting information

S1 Fig Representative histological images of scored findings. Examples of 14 scored pathological features judged negative (left side) and positive (right side). Negative example of lymphoid follicle with germinal center is simply a normal lung tissue and is not included in this file. HC, honeycomb; FF, fibroblastic focus; SMH, smooth muscle hyperplasia; OP, organising pneumonia; CIP, cellular interstitial pneumonia; Plasm, prominent plasmacytic infiltration; LyGC, lymphoid follicle with germinal center; PLE, extensive pleuritis; VT, vascular intimal thickening; DPVC, dense perivascular collagen; AF, airspace fibrin; Fat, fat metaplasia; CB, constrictive bronchiolitis.
(PPTX)

S2 Fig Kaplan Meier curve of IIP and CTD-IP. The derivation cases had follow up data, and Kaplan Meier curve was plotted. There is no significant survival difference between CTD-IP and IIP in this group.
(PPTX)

S3 Fig Creation of web-based application (APP). The app is composed of 8 pages of queries for histological findings (A). The final page shows suggested diagnosis and likelihood of the diagnosis based on the probability: A, 1.0 to 0.8 and 0.19 to 0; B, 0.79 to 0.6 and 0.39 to 0.2; C, 0.59 to 0.4 (B).
(PPTX)

S1 Table Autoantibodies of the cases. (XLSX)
