# Supplementary material for: Proposed method of histological separation between connective tissue disease-associated interstitial pneumonia and idiopathic interstitial pneumonias
Source: PLoS One. 2018 Nov 5;13(11):e0206186. doi: 10.1371/journal.pone.0206186 (PMC6218032; doi:10.1371/journal.pone.0206186)
Supplement: S1 Fig — Examples of 14 scored pathological features judged negative (left side) and positive (right side). Negative example of lymphoid follicle with germinal center is simply a normal lung tissue and is not included in this file. HC, honeycomb; FF, fibroblastic focus; SMH, smooth muscle hyperplasia; OP, organising pneumonia; CIP, cellular interstitial pneumonia; Plasm, prominent plasmacytic infiltration; LyGC, lymphoid follicle with germinal center; PLE, extensive pleuritis; VT, vascular intimal thickening; DPVC, dense perivascular collagen; AF, airspace fibrin; Fat, fat metaplasia; CB, constrictive bronchiolitis. (PPTX) [file pone.0206186.s001.pptx]

## Slide 1
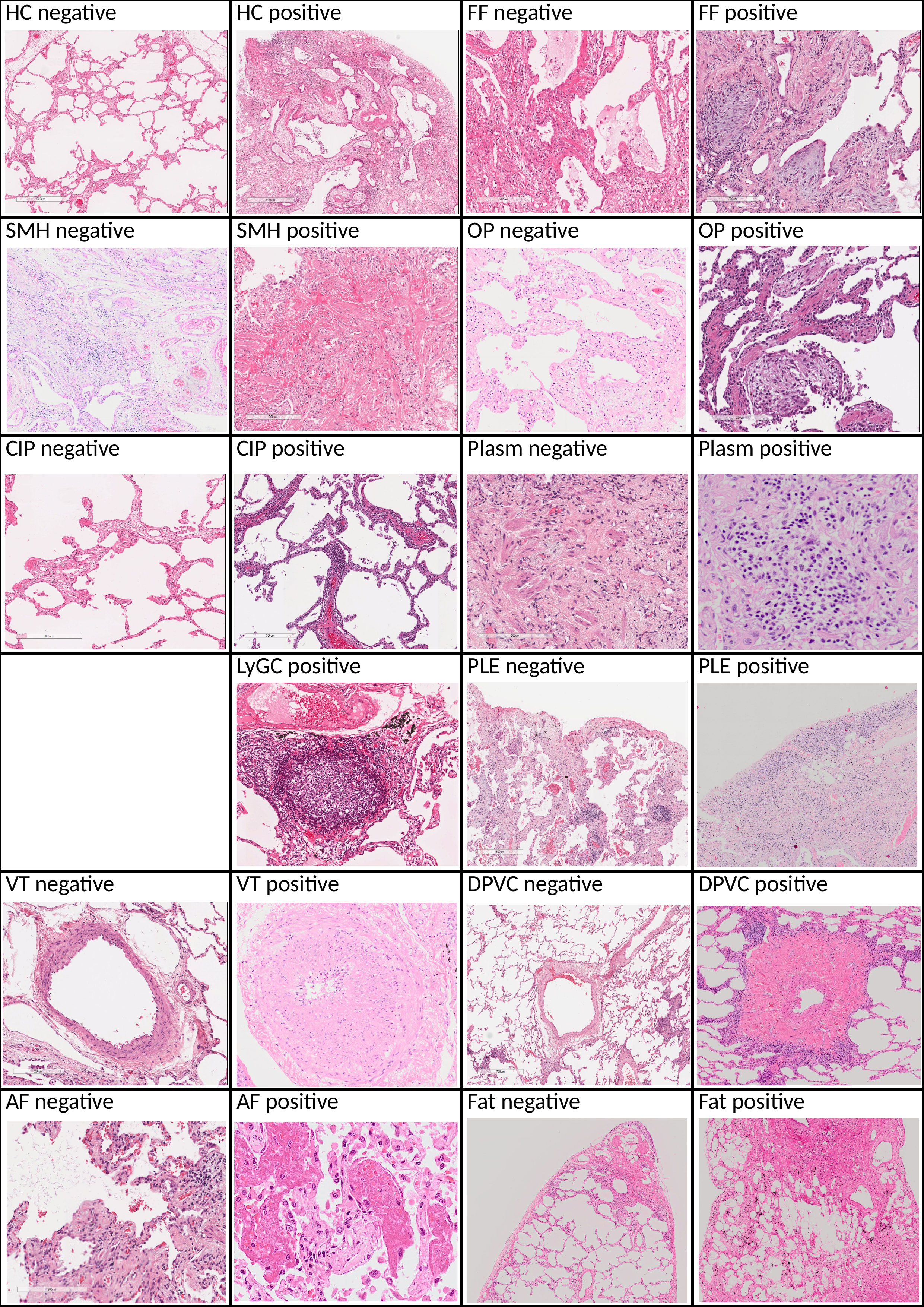

| HC negative | HC positive | FF negative | FF positive |
| --- | --- | --- | --- |
| SMH negative | SMH positive | OP negative | OP positive |
| CIP negative | CIP positive | Plasm negative | Plasm positive |
| | LyGC positive | PLE negative | PLE positive |
| VT negative | VT positive | DPVC negative | DPVC positive |
| AF negative | AF positive | Fat negative | Fat positive |

## Slide 2
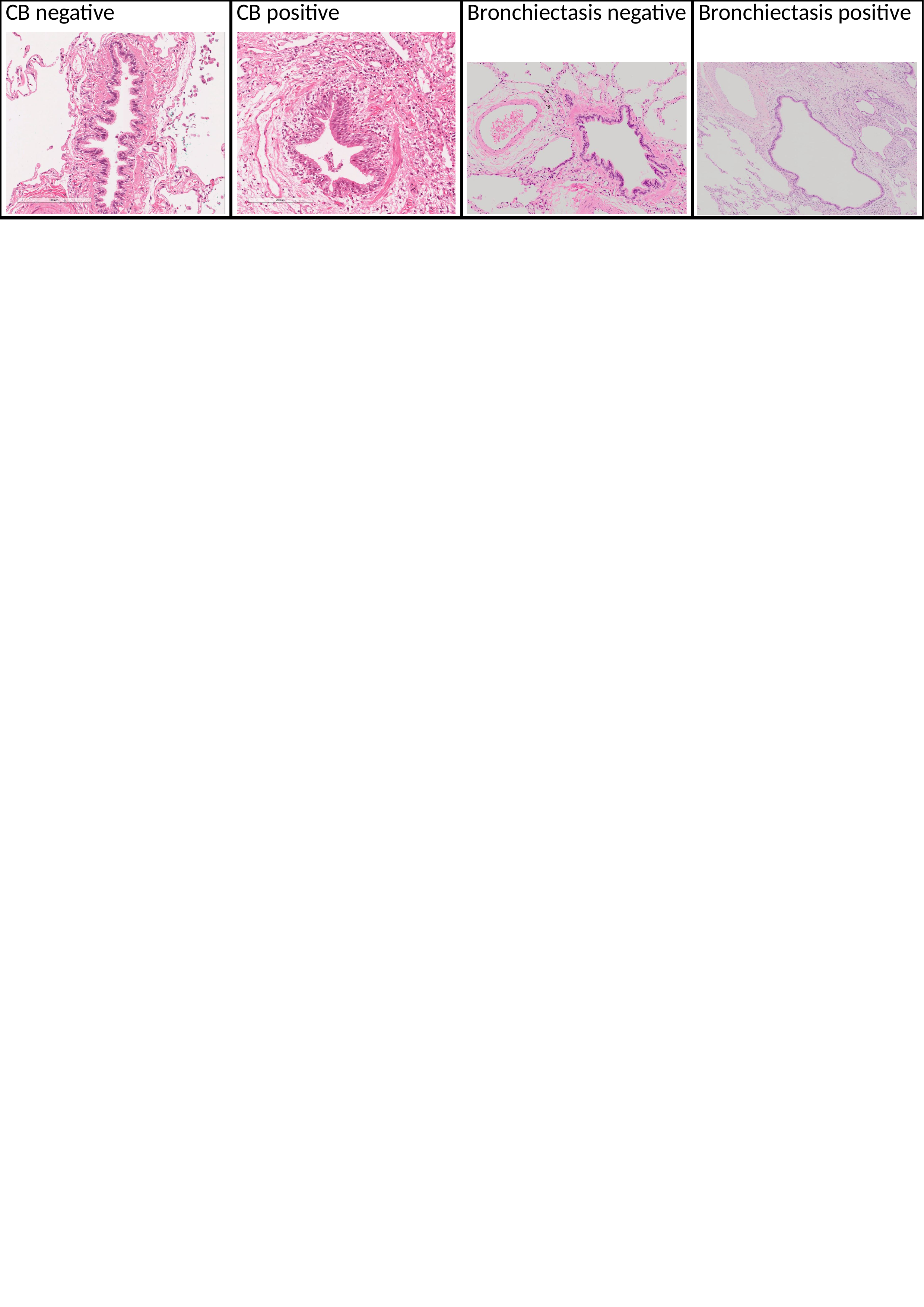

| CB negative | CB positive | Bronchiectasis negative | Bronchiectasis positive |
| --- | --- | --- | --- |
