# Supplementary figures and images for: Proposed method of histological separation between connective tissue disease-associated interstitial pneumonia and idiopathic interstitial pneumonias
Source: PLoS One. 2018 Nov 5;13(11):e0206186. doi: 10.1371/journal.pone.0206186 (PMC6218032; doi:10.1371/journal.pone.0206186)

## Slide 1
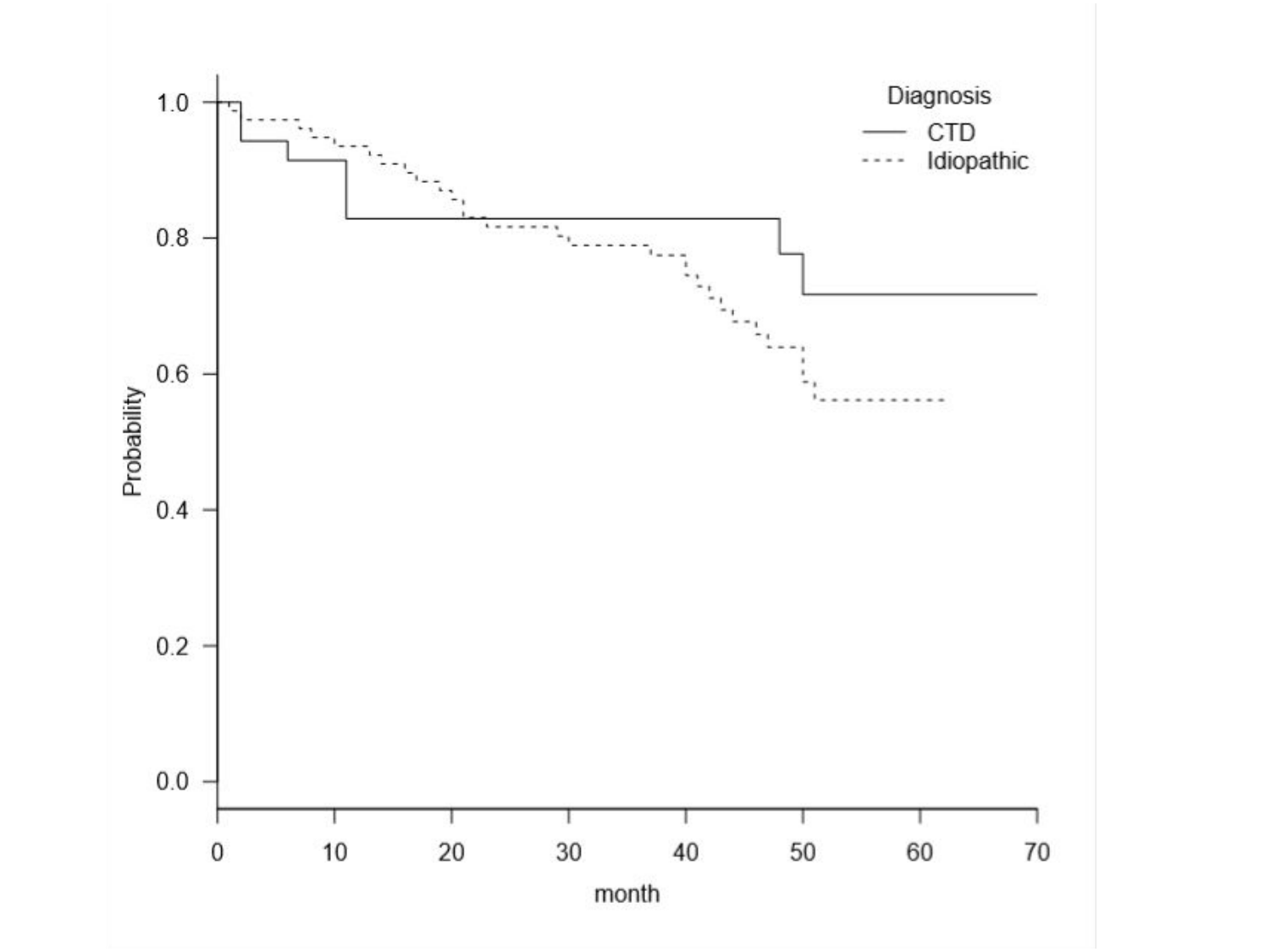

Supplement: S2 Fig — The derivation cases had follow up data, and Kaplan Meier curve was plotted. There is no significant survival difference between CTD-IP and IIP in this group. (PPTX) [file pone.0206186.s002.pptx]

## Slide 1
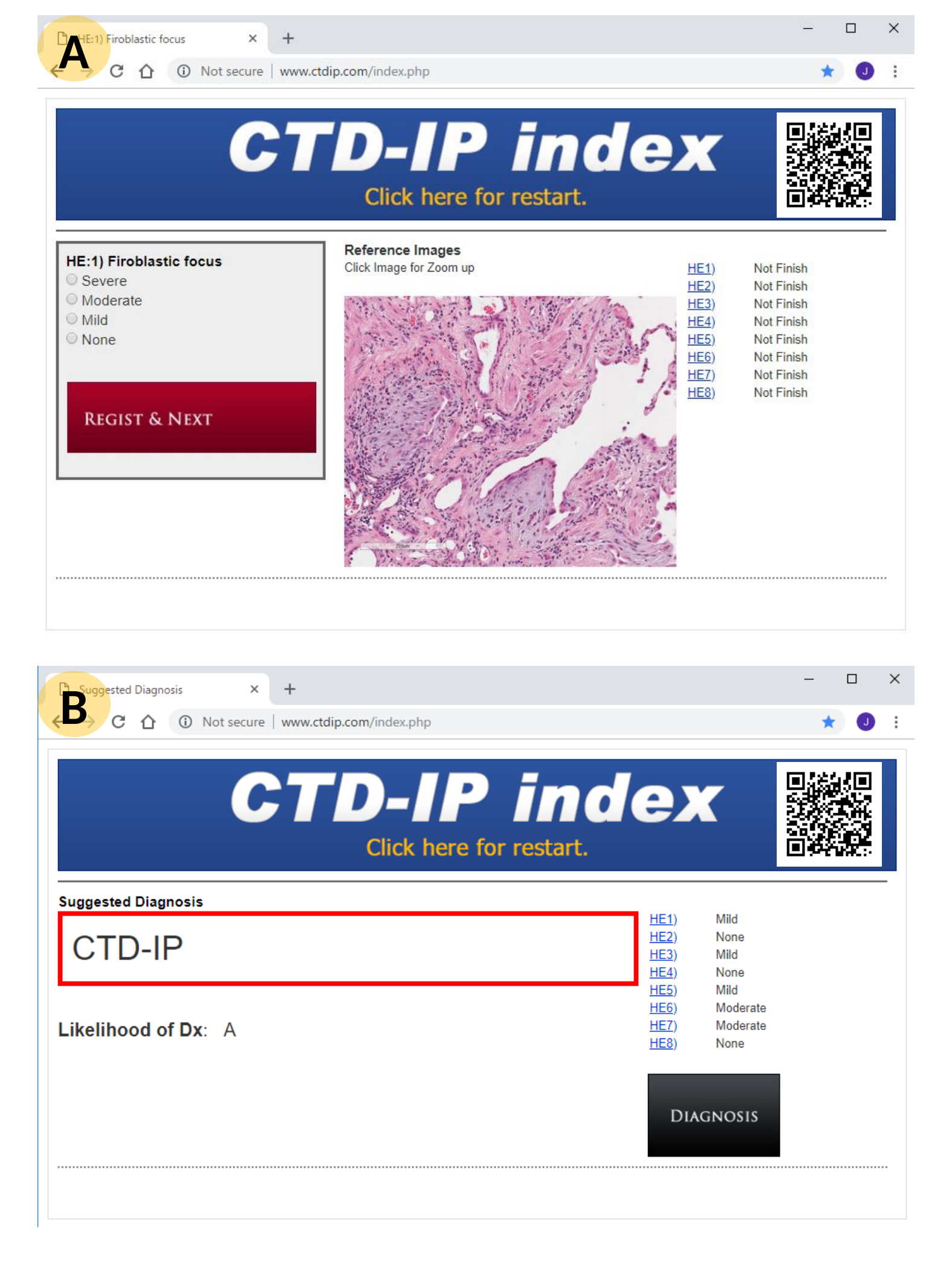

A
B

Supplement: S3 Fig — The app is composed of 8 pages of queries for histological findings (A). The final page shows suggested diagnosis and likelihood of the diagnosis based on the probability: A, 1.0 to 0.8 and 0.19 to 0; B, 0.79 to 0.6 and 0.39 to 0.2; C, 0.59 to 0.4 (B). (PPTX) [file pone.0206186.s003.pptx]
